# Influence of Dose Rate on the Cellular Response to Low- and High-LET Radiations

**DOI:** 10.3389/fonc.2016.00058

**Published:** 2016-03-14

**Authors:** Anne-Sophie Wozny, Gersende Alphonse, Priscillia Battiston-Montagne, Stéphanie Simonet, Delphine Poncet, Etienne Testa, Jean-Baptiste Guy, Chloé Rancoule, Nicolas Magné, Michael Beuve, Claire Rodriguez-Lafrasse

**Affiliations:** ^1^UMR/CNRS 5822, Laboratoire de Radiobiologie Cellulaire et Moléculaire, Université Claude Bernard Lyon 1, Oullins, France; ^2^Centre Hospitalier Lyon-Sud, Hospices-Civils-de-Lyon, Pierre-Bénite, France; ^3^IPNL-LIRIS-CNRS-IN2P3, Villeurbanne, France; ^4^Département de Radiothérapie, Institut de Cancérologie de la Loire Lucien Neuwirth, St-Priest-en-Jarez, France

**Keywords:** high- and low-LET irradiations, carbon ions, photons, dose rate, DNA double-strand breaks, head and neck squamous cell carcinoma

## Abstract

Nowadays, head and neck squamous cell carcinoma (HNSCC) treatment failure is mostly explained by locoregional progression or intrinsic radioresistance. Radiotherapy (RT) has recently evolved with the emergence of heavy ion radiations or new fractionation schemes of photon therapy, which modify the dose rate of treatment delivery. The aim of the present study was then to evaluate the *in vitro* influence of a dose rate variation during conventional RT or carbon ion hadrontherapy treatment in order to improve the therapeutic care of patient. In this regard, two HNSCC cell lines were irradiated with photons or 72 MeV/n carbon ions at a dose rate of 0.5, 2, or 10 Gy/min. For both radiosensitive and radioresistant cells, the change in dose rate significantly affected cell survival in response to photon exposure. This variation of radiosensitivity was associated with the number of initial and residual DNA double-strand breaks (DSBs). By contrast, the dose rate change did not affect neither cell survival nor the residual DNA DSBs after carbon ion irradiation. As a result, the relative biological efficiency at 10% survival increased when the dose rate decreased. In conclusion, in the RT treatment of HNSCC, it is advised to remain very careful when modifying the classical schemes toward altered fractionation. At the opposite, as the dose rate does not seem to have any effects after carbon ion exposure, there is less need to adapt hadrontherapy treatment planning during active system irradiation.

## Introduction

Head and neck cancer is the sixth most common type of cancer worldwide. More than 600,000 new patients are diagnosed per year among which approximately 350,000 will die (1). Over 60% of patients present a locally advanced stage (III–IV) at diagnosis, and despite improvements in the therapeutic management of this type of cancer in recent years, disease-free survival at 3 years does not exceed 30% and overall survival at 5 years is less than 50% (2). Three major therapeutic strategies are used for non-operated stage III and IV squamous cell carcinoma: (1) combined radiotherapy (RT) and concomitant platin-based chemotherapy (CT), (2) concomitant association of RT with cetuximab (anti-EGFR), and (3) induction CT with a combination of docetaxel, cisplatin, and 5-fluorouracil (TPF) followed by RT alone (3). If high response rates are observed after TPF ± RT (68 and 72%, respectively), with 8.5 and 17% of complete response (3, 4), disease-free survival remains poor. Recurrences are mainly related to a locoregional progression (85 and 86%), and this failure could be linked to an acquired or inherent radioresistance to low-LET radiation.

Improvements in RT over the last decade are linked to the emergence of intensity-modulated techniques and new protocols of altered fractionation, which modify the dose rate of treatment delivery or to the use of heavy ion species (5). Hyperfractionation shows improvements in survival of head and neck squamous cell carcinoma (HNSCC) patients (6) and hypofractionated schemes are efficient for palliation (7). However, limited biological data have up to now been published on the effect of a dose rate variation on the tumor survival. In parallel, hadrontherapy offers advantages over conventional RT due to the physical and biological properties of carbon ion irradiation, which have an enhanced radiobiological effectiveness (RBE) caused by the dense ionization, resulting in complex irreparable DNA lesions (8). Therefore, hadrontherapy is a very promising approach for treating radioresistant tumors located near organs at risk (9). However, carbon ions delivered by an active scanning system cause an important variation in the dose rate within the tumor volume. The deepest parts of the tumor are irradiated in less than 1 s, whereas the shallowest parts of the tumor are irradiated in a few minutes. Moreover, modern techniques of photon radiation, such as intensity-modulated RT, may increase the number of radiation fields, and thus the dose is delivered to the tumor in a longer time. If cellular radiosensitivity is dose rate dependent after photon exposure, very little is known about high-LET radiation dose rate response.

The aim of this work was then to better understand the effects of doses’ variations for high- and low-LET radiations on cancer cell lines in terms of radiosensitivity, cell survival, and DNA double-strand-breaks in order to better adapt the radio- and hadrontherapy treatment.

## Materials and Methods

### Cell Culture

Two HNSCC cell lines were used. The radiosensitive SCC61 and the radioresistant SQ20B cell lines were grown, as previously described (10, 11).

### Irradiation Procedure

Photon irradiations were performed on a 250-kV irradiator (X-RAD 320, PXI), at the Lyon-Sud University (11) (UMS3444/US8 platform, France), and carbon ion irradiations (72 MeV/n, LET 33.6 keV/μm), at Grand Accélérateur National d’Ions Lourds (GANIL, Caen, France), facilities, as previously described (12, 13).

### Analysis of Clonogenic Cell Survival

Cell survival following irradiation was quantified using a colony forming assay, as previously described (12, 14). Ten to sixteen hours before irradiation, SCC61 and SQ20B cells were seeded in 25 cm^2^ flasks at different densities, depending on the dose of radiation. Cells were irradiated at room temperature at 1, 2, 3, 4, or 5 Gy delivered at a dose rate of 0.5, 2, or 10 Gy/min, respectively. For each dose and dose rate tested, six flasks were irradiated at two different cell densities. After irradiation, flasks were replaced in the incubator at 37°C. After six cellular divisions, colonies were fixed with ethanol 95% and stained with Giemsa (1/20). The number of colonies containing at least 64 cells was counted using Coltcount (Optronix), and the surviving fractions were calculated using the formula *S*(*D*) = *n*(*D*)/PE × *N*(*D*), where *n* represents the number of colonies, *N* the seeded cell number, and PE the plating efficiency. Each experiment was realized in triplicate.

### Immunocytochemistry – γH2AX Assay

The method was adapted from Tanaka et al. (15). Cells were irradiated at 1 and 2 Gy with photons or carbon ions at a dose rate of 0.5, 2, or 10 Gy/min. At 15 min (only for photons), 30 min, 1, 2, and 24 h after irradiation, cells were trypsinized, washed with PBS, and fixed in 70% ice-cold ethanol for at least 24 h. Cells were then resuspended in PBS for a wash and incubated in permeabilization buffer (20 mM HEPES, 50 mM NaCl, 3 mM MgCl_2_, 300 mM sucrose, and 0.5% Triton X-100 in PBS). After two washes in PBSMB (PBS 1% milk, 0.1% BSA), cells were incubated for 2 h with gentle agitation in a primary antibody solution consisting of an antiphospho-histone-H2AX (serine139) mouse monoclonal IgG1 antibody (Millipore, Watford, UK) diluted at 1/2000 in PBSMB. Excess primary antibody was removed by washing twice in PBSMB buffer. A secondary antibody solution consisting of Alexa Fluor-488 goat-antimouse IgG antibody (Invitrogen) diluted at 1/1000 in blocking buffer was added to each sample and incubated for 20 min at room temperature. Excess secondary antibody was removed by washing twice with PBSMB. Cells were finally resuspended in PBS for flow cytometry analysis. A minimum of 10,000 cells were analyzed using a FACS-BD-LSRII.

### Statistical Analysis

Statistical analyses were performed using the R software. The two-way ANOVA statistical test was used to compare the interaction between the dose and the dose rate in order to determine the significance of the differences (a *p*-value <0.05 was considered statistically significant). The Student’s *t*-test was also used to compare values between groups.

## Results

### Influence of Dose Rate Variation on HNSCC Radiosensitivity after High- and Low-LET Exposure

**Figure 1:**
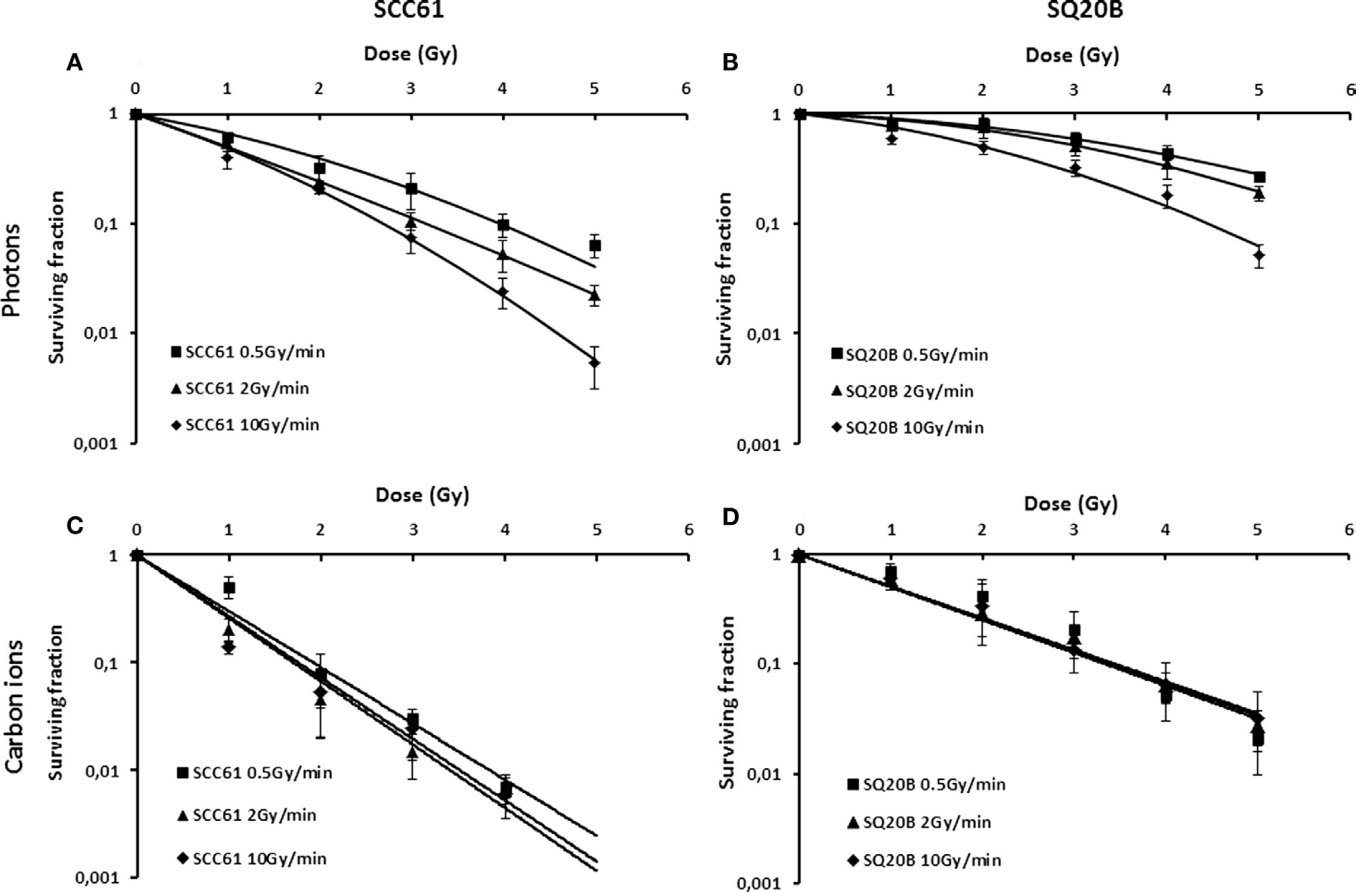
**Dose–response curves for killing of SCC61 (A,C) and SQ20B cells (B,D) in response to photon (A,B) or 72 MeV/n carbon ion (C,D) irradiation at a dose rate of 0.5 Gy/min (full square), 2 Gy/min (full triangle), or 10 Gy/min (full diamonds)**. Values represent the mean ± SD of three independent experiments performed in sextuplicate.

Figure 1 shows the dose–response curves for cell killing induction in the radiosensitive SCC61 and radioresistant SQ20B cells after exposure to both carbon ion beams and photons. In response to photon irradiation, a significant change in the survival fraction at 2 Gy (SF2) and the dose for 10% survival (D10) was observed for both cell lines depending on the dose rate. For SCC61 cells, the SF2 obtained after a 0.5 Gy/min photon irradiation was 0.39, whereas it significantly fell to 0.24 after a 2 Gy/min and to 0.20 after a 10 Gy/min irradiation. When statistical analysis was done, a significant difference (*p* = 0.02) between the three dose rate survival curves was observed. The same variation of the SF2 was observed for the radioresistant cell line SQ20B, where the SF2 value changed from 0.76 after a 0.5 Gy/min photon irradiation to 0.71 after a 2 Gy/min irradiation and to 0.50 after a 10 Gy/min irradiation (Table 1). A significant difference (*p* = 0.04) between the different dose rate survival curves was also observed.

**Table 1:** **Radiobiological parameters of SCC61 and SQ20B cell lines for a 0.5, 2, or 10 Gy/min photon or carbon ion irradiation**.

	Dose rate (Gy/min)	SF2 photons	SF2 carbon ions	D10 photons	D10 carbon ions	RBE
SCC61	0.5	0.39	0.09	3.9	1.9	2.1
	2	0.24	0.07	3.1	1.7	1.8
	10	0.20	0.07	2.7	1.7	1.6
SQ20B	0.5	0.76	0.26	6.9	3.4	2.0
	2	0.71	0.26	6.0	3.4	1.8
	10	0.50	0.26	4.4	3.4	1.3

*SF2, survival fraction at 2 Gy; D10, dose for 10% survival; RBE, relative biological effect of carbon ions at 10% survival*.

The survival curves realized after carbon ion irradiation were fitted either using simple linear or linear quadratic fit curves. Whatever the fit considered, and conversely to photon irradiation, dose rate changes in response to carbon ion irradiation did not affect the radiosensitivity. Whatever the radiosensitivity of the cells, the variation of the dose rate did not cause any change in cell survival, SF2, or D10 values. For SQ20B cells, the SF2 is 0.26 whatever the dose rate considered. For the SCC61 cell line, SF2 varies from 0.09 to 0.07 depending on the dose rate (*p* = 0.21 for the three survival curves with linear fit and *p* = 0.14 for linear quadratic fit).

From these results, the RBE at 10% survival was calculated (Table 1). The RBE increased when the dose rate decreased both in the radiosensitive and radioresistant cell lines. For a dose rate of 0.5 Gy/min, RBE values are 2.1 and 2.0 for SCC61 and SQ20B cells, respectively; and they fall to 1.6 and 1.3 when a dose rate of 10 Gy/min is applied.

### DNA Double-Strand-Break Analysis by γH2AX Flow Cytometry Assay

**Figure 2:**
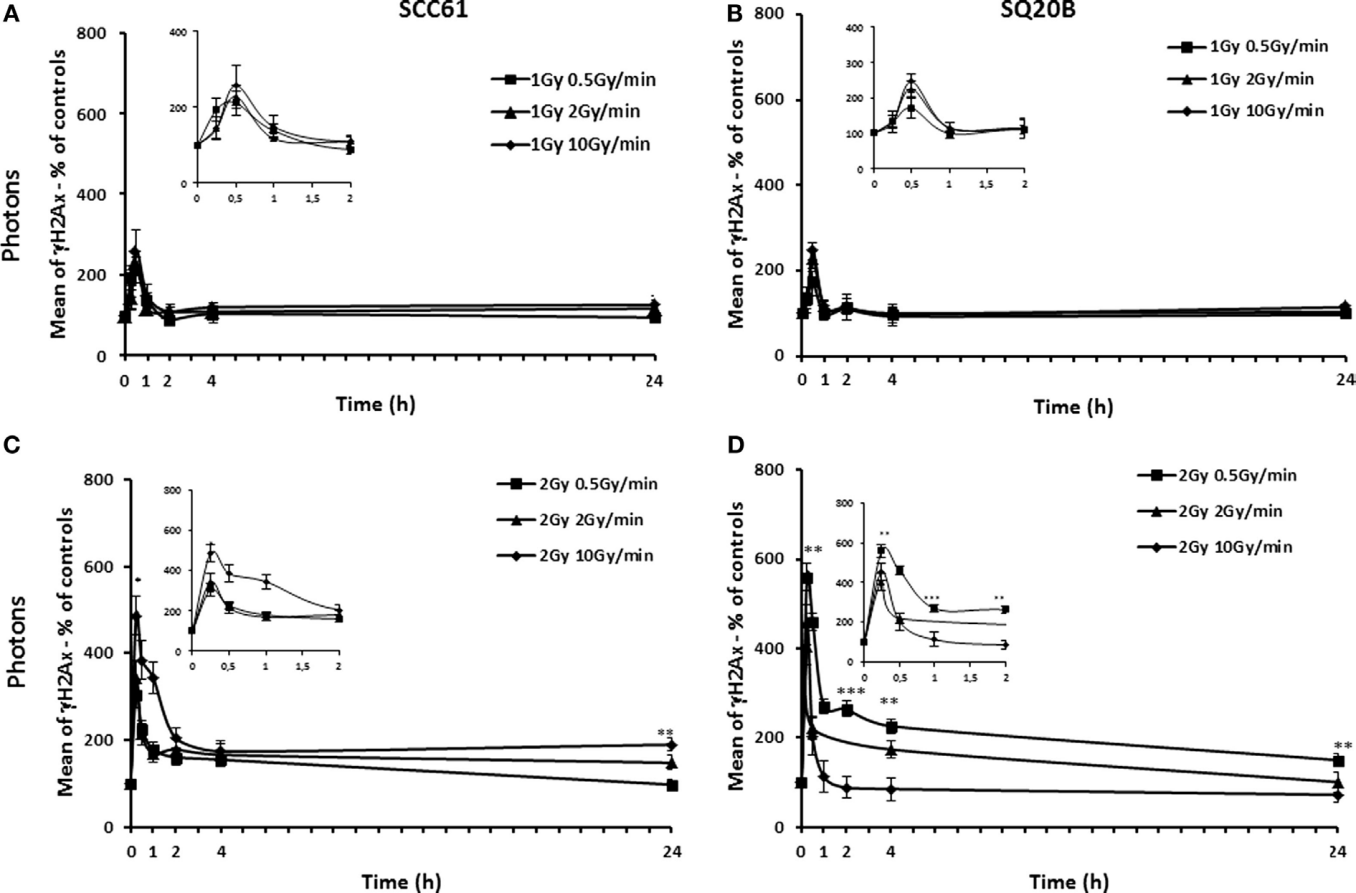
**Kinetic study of γH2AX foci**. SCC61 **(A,C)** and SQ20B cells **(B,D)** were irradiated with 1 or 2 Gy photons at a dose rate of 0.5 Gy/min (full square), 2 Gy/min (full triangle), or 10 Gy/min (full diamonds). Short time points are represented in the insert. The percentage of γH2AX foci was calculated using sham-irradiated cells. For each time point, 10,000 cells were analyzed by flow cytometry. Values represent the mean ± SD of one or two independent experiments performed in triplicate. *p < 0.05, **p < 0.01, and ***p < 0.001.

**Figure 3:**
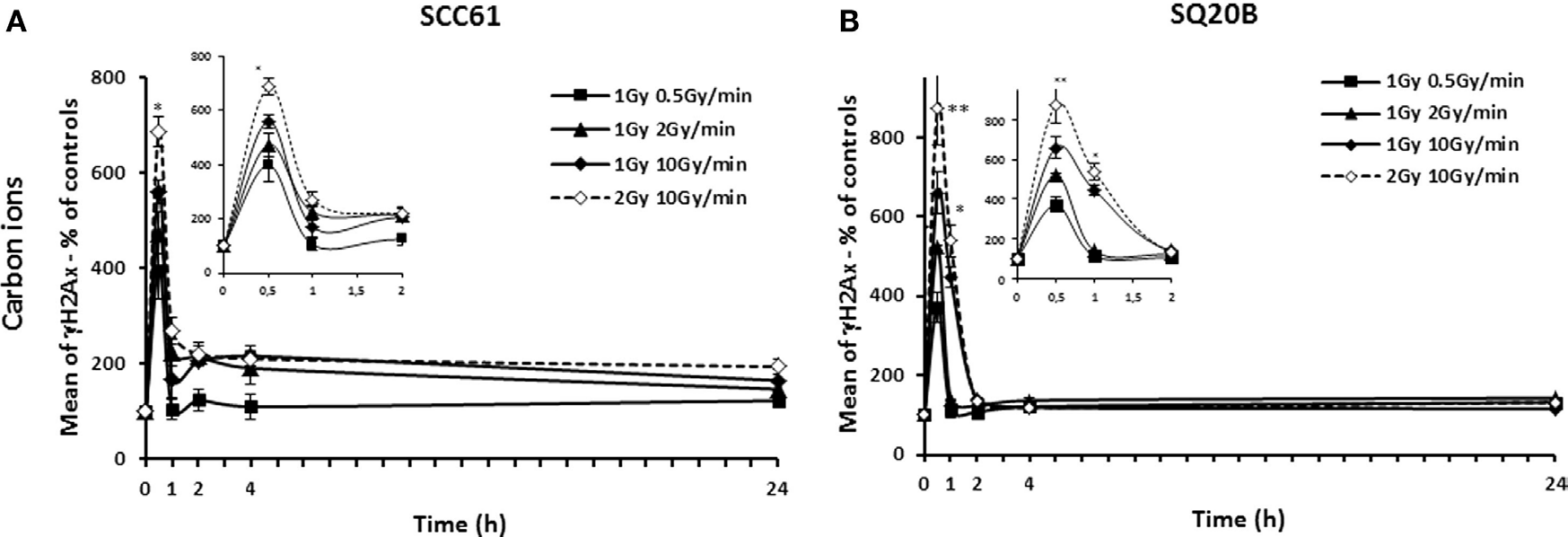
**Kinetic study of γH2AX foci**. SCC61 **(A)** and SQ20B cells **(B)** were irradiated with 1 Gy carbon ions at a dose rate of 0.5 Gy/min (full square), 2 Gy/min (full triangle), or 10 Gy/min (full diamonds). A kinetic study at 10 Gy/min for 2 Gy carbon ions is represented in dotted line. Short time points are represented in the insert. The percentage of γH2AX foci was calculated using sham-irradiated cells. For each time point, 10,000 cells were analyzed by flow cytometry. Values represent the mean ± SD of two independent experiments performed in triplicate.

Figures 2 and 3 show the γH2AX fluorescence for both cell lines after photon or carbon ion exposure reported to that of sham-irradiated controls. Different kinetic studies, from 15 min to 24 h, were realized after 1 and 2 Gy photon or carbon ion exposure in order to compare the effect for the same physical dose (1 Gy photons compared to 1 Gy carbon ions and 2 Gy photons compared to 2 Gy carbon ions) as well as the effect for the same biological equivalent dose (1 Gy carbon ions compared to 2 Gy photons). The biological equivalent dose was calculated using an RBE of 2, previously calculated for a dose rate of 2 Gy/min (12).

At equivalent biological dose of photons and carbon ions, the initial peak of γH2AX foci, obtained 30 min after irradiation, was almost similar in both the radioresistant SQ20B and the radiosensitive SCC61 cells when the same dose rate was considered. At the opposite, for a same physical dose and a same dose rate, the initial peak for both cell lines was higher after a carbon ion irradiation compared with photon irradiation. However, whatever the dose or the type of irradiation is, the intensity of the peak depended on the dose rate. As an example, for the SQ20B cells, 30 min after 1 Gy of carbon ions, the percentage of γH2AX fluorescence was 370 ± 39% compared to the sham-irradiated control for a dose rate of 0.5 Gy/min, 521 ± 26% for 2 Gy/min, and 658 ± 52% for 10 Gy/min. For all types of radiations and cells, the decrease of the amount in γH2AX took longer time for the higher dose rate. If we consider the residual double-strand breaks (DSBs) 24 h after irradiation, the results differed between the types of irradiation for both cell lines. Indeed, an effect of the dose rate was obtained after photon irradiation, whereas none was found after carbon ion irradiation (1 or 2 Gy). For both SCC61 and SQ20B irradiated with 2 Gy photons, a higher percentage of residual γH2AX fluorescence was found at a dose rate of 10 Gy/min (190 ± 15 and 148 ± 16%, respectively) compared to 150 ± 11 and 98 ± 12%, respectively, when the dose rate decreases to 2 Gy/min. After a high-LET irradiation, the responses of both cell lines were different. For the radiosensitive cells, the residual number of DNA DSBs varied between 165 ± 23 and 123 ± 13% after 10 and 0.5 Gy/min, respectively, but this variation was not statistically significant. For the radioresistant cell line, the percentage of residual γH2AX foci was lower, between 132 ± 11 and 115 ± 3 for 10 and 0.5 Gy/min, respectively, and also not significant.

## Discussion

Our study demonstrates that a variation in the dose rate affects cell survival only for low-LET irradiation and that this variation does not depend on the initial radiosensitivity of the cell line considered. Although the effects of an important dose rate variation (gray per second to gray per hour) after low-LET radiation has been reported for several years (16), very few studies related these effects in regard to the intensity-modulated RT techniques or in response to high-LET irradiation. Therefore, the aim of our work was to study the effect of a dose rate variation during high- or low-LET irradiations in terms of cell survival and DNA DSB repair. Our study was realized using two HNSCC cell lines displaying opposite radiosensitivity, irradiated with photons or carbon ions at dose rates of 0.5, 2, or 10 Gy/min. These dose rates correspond to an irradiation time varying from 15 s to 20 min, matching with the irradiation duration associated with the active ion beam techniques, on one hand, and, to the sessions of multifield irradiation, on the other hand.

First of all, we confirmed previous data (16, 17) demonstrating that even with a very slight change in the dose rate of photon, a variation of radiosensitivity (SF2 and D10) was observed. Moreover, this variation does not depend on the intrinsic radiosensitivity. In response to photon irradiation, it has been previously reported that a significant reduction in the lethality, mutagenesis, and carcinogenesis was observed when the dose is delivered at low dose rates compared to high dose rates [for review, see Ref. (18)]. The advanced hypothesis to explain these results was that damage spreads more over a time at a low dose rate, which should allow cells to have more time to detect DNA damages and activate their repair systems (19). Our results obtained for both cell lines confirm this hypothesis and are in total accordance with our previous published study (20), which reported that more initial and residual γH2AX foci were found after photon irradiation. Boucher et al. (21) have also demonstrated that low-LET radiation-induced lethality at low dose rate was accompanied by a decreased number of DSBs. They have additionally highlighted the involvement of the non-homologous end joining pathway in the repair of gamma-induced DSBs at 20 mGy/min. The impact of the dose rate was also studied on the induction of apoptotic cell death. In lymphocyte, it appears that only a very low dose rate has an effect on the induction of apoptosis (22). This is of particular interest in head and neck locations, where intensity-modulated techniques are used, and dose rate variations are observed in the different planned beams. Since static intensity-modulated-RT is the gold standard for head and neck RT (23), this technique increases the treatment time compared to 3D conformational RT. Volumetric modulated arc therapy has thus been developed and reduced by 50% treatment time (24). This technique can limit intrafraction patients’ movements and maintain an acceptable dose rate through the decreased treatment time. In our study, dose rate influence in photon irradiation suggests that the radiation therapist has to take it into account when choosing the planning technique.

As no data, obtained in the same conditions, are up to now available with high-LET, the second important aim of this study was to evaluate the influence of a high-LET dose rate variation on HNSCC cells. We demonstrated that, in contrast to photon irradiation, a carbon ion dose rate variation did not significantly affect cell survival irrespective of whatever the intrinsic radio sensitivity of the cell line used. As a consequence, we have demonstrated that the RBE at 10% survival increased when the dose rate decreased. Our results are in accordance with Masunaga et al. (25) who have studied the RBE of 290 MeV/n carbon ions, a LET found in the healthy tissues, by changing only the photon dose rate in quiescent tumor cells. Unlike us, the LET used in this study correspond to that found at the beginning of the SOBP. In order to confirm our results, it would now be of great interest to test the influence of a dose rate variation with a higher LET corresponding to that found in the center of the tumor. Moreover, as experiments were done only in tumor cells, it would be essential to study the response of healthy tissue surrounding the tumor.

We also evaluated DNA damage in our work. Although there is a slight effect on initial DNA DSBs 30 min after carbon ion irradiation, depending on the dose rate, no difference was observed on the residual ones. It is now well established that a localized deposition of high-LET particle results in complex DNA DSBs that cause cell death, mutations, and genomic instability (20, 26). By contrast, the repair mechanisms of high-LET-induced DSBs are not fully understood (27). From our results, we can assume that the DNA damage response processes seems to be different between photon and carbon ion irradiation and then complementary experiments need to be done, in order to explain why there is no effect of the dose rate on cell survival after high-LET irradiation.

In conclusion, this study was carried out to understand whether a variation of the dose rate during a photon altered fractionation, intensity-modulated-RT, or a hadrontherapy treatment could affect cell survival or not. This study was of crucial interest since during hadrontherapy there is an important variation of the dose rate occurring within the tumor and since the adaptation of current HNSCC RT treatment schemes and techniques is still performed empirically. Thus, we have shown that in response to photon exposure, the change in dose rate significantly affected cell survival for both radiosensitive and radioresistant cells, this variation of radiosensitivity was directly correlated to the number of initial and residual DNA DSBs. By contrast, the dose rate change did not affect neither cell survival nor the residual DNA DSBs after carbon ion irradiation.

## Author Contributions

Substantial author contributions: to the conception or design of the work: GA, MB, and CR-L; to the acquisition: GA, A-SW, PB-M, SS, and ET; to the analysis: GA, A-SW, and J-BG; to the interpretation: DP, A-SW, GA, CR, CR-L, and MB; and to the final approval of the manuscript: NM and CR-L.

## Conflict of Interest Statement

The authors declare that the research was conducted in the absence of any commercial or financial relationships that could be construed as a potential conflict of interest.
