# Corrigendum: Influence of Dose Rate on the Cellular Response to Low- and High-LET Radiations

**DOI:** 10.3389/fonc.2016.00271

**Published:** 2017-01-19

**Authors:** Anne-Sophie Wozny, Gersende Alphonse, Priscillia Battiston-Montagne, Stéphanie Simonet, Delphine Poncet, Etienne Testa, Jean-Baptiste Guy, Chloé Rancoule, Nicolas Magné, Michael Beuve, Claire Rodriguez-Lafrasse

**Affiliations:** ^1^UMR/CNRS 5822, Laboratoire de Radiobiologie Cellulaire et Moléculaire, Univ Lyon, UCBL1, Oullins, France; ^2^Centre Hospitalier Lyon-Sud, Hospices-Civils-de-Lyon, Pierre-Bénite, France; ^3^IPNL-LIRIS-CNRS-IN2P3, Villeurbanne, France; ^4^Departement de Radiothérapie, Institut de Cancérologie de la Loire Lucien Neuwirth, St-Priest-en-Jarez, France

**Keywords:** high- and low-LET irradiations, carbon ions, photons, dose rate, DNA double-strand breaks, head-and-neck squamous cell carcinoma

The error is in the Materials and Methods section. It concerns the energy of the irradiation and the type of irradiator used. The correct version of sub-section “Irradiation Procedure” of the Materials and Methods section appears below. The authors sincerely apologize for the mistake. This error does not change the scientific conclusions of the article in any way.

## Irradiation Procedure

Photon irradiations were performed in the radiation therapy department of Hospices Civils de Lyon (France) using a Clinac CD linear accelerator (Varian Medical Systems, Inc., Palo Alto, CA, USA) and Carbon ion irradiations (72 MeV/u, LET 33.6 keV/µm) were realized at GANIL (Grand Accélérateur National d’Ions Lourds, Caen, France) facilities as previously described (1, 2).

## Conflict of Interest Statement

The authors declare that the research was conducted in the absence of any commercial or financial relationships that could be construed as a potential conflict of interest.
